# Heterogeneous root zone salinity mitigates salt injury to *Sorghum bicolor* (L.) Moench in a split-root system

**DOI:** 10.1371/journal.pone.0227020

**Published:** 2019-12-30

**Authors:** Huawen Zhang, Runfeng Wang, Hailian Wang, Bin Liu, Mengping Xu, Yan’an Guan, Yanbing Yang, Ling Qin, Erying Chen, Feifei Li, Ruidong Huang, Yufei Zhou

**Affiliations:** 1 Agronomy College, Shenyang Agricultural University, Shenyang, Liaoning, China; 2 Crop Research Institute, Shandong Academy of Agricultural Sciences, Jinan, Shandong, China; 3 Shandong Engineering Laboratory for Featured Crops, Jinan, Shandong, China; Rutgers The State University of New Jersey, UNITED STATES

## Abstract

The heterogeneous distribution of soil salinity across the rhizosphere can moderate salt injury and improve sorghum growth. However, the essential molecular mechanisms used by sorghum to adapt to such environmental conditions remain uncharacterized. The present study evaluated physiological parameters such as the photosynthetic rate, antioxidative enzyme activities, leaf Na^+^ and K^+^ contents, and osmolyte contents and investigated gene expression patterns via RNA sequencing (RNA-seq) analysis under various conditions of nonuniformly distributed salt. Totals of 5691 and 2047 differentially expressed genes (DEGs) in the leaves and roots, respectively, were identified by RNA-seq under nonuniform (NaCl-free and 200 mmol·L^-1^ NaCl) and uniform (100 mmol·L^-1^ and 100 mmol·L^-1^ NaCl) salinity conditions. The expression of genes related to photosynthesis, Na^+^ compartmentalization, phytohormone metabolism, antioxidative enzymes, and transcription factors (TFs) was enhanced in leaves under nonuniform salinity stress compared with uniform salinity stress. Similarly, the expression of the majority of aquaporins and essential mineral transporters was upregulated in the NaCl-free root side in the nonuniform salinity treatment, whereas abscisic acid (ABA)-related and salt stress-responsive TF transcripts were more abundant in the high-saline root side in the nonuniform salinity treatment. In contrast, the expression of the DEGs identified in the nonuniform salinity treatment remained virtually unaffected and was even downregulated in the uniform salinity treatment. The transcriptome findings might be supportive of the increased photosynthetic rate, reduced Na^+^ levels, increased antioxidative capability in the leaves and, consequently, the growth recovery of sorghum under nonuniform salinity stress as well as the inhibited sorghum growth under uniform salinity conditions. The increased expression of salt resistance genes activated in response to the nonuniform salinity distribution implied that the cross-talk between the nonsaline and high-saline sides of the roots exposed to nonuniform salt stress is potentially regulated.

## Introduction

Crop yields are significantly challenged by an extensive range of environmental constraints. One of the major performance-limiting factors is soil salinity [1]. Approximately 80 million hectares of global agricultural land have been salinized [2]. Even worse, inappropriate human production and agricultural activities have been exacerbating soil salinization by 10% annually, at which pace more than half of the global cultivated land today will be affected by soil salinity by 2050 [3]. High soil salt concentrations can cause osmotic stress and ionic toxicity, which result in slowed growth, reduced productivity, dysfunctional metabolism and excess production of reactive oxygen species (ROS) due to water/nutrient uptake deficiencies, and disturbed Na^+^/K^+^ ratios [3–5]. To combat high soil salinity, plants have evolved many endogenous mechanisms to alleviate the deleterious effects of salt damage and water/nutrient deficiency.

Plants respond to Na^+^ injury and maintain ionic homeostasis by either extruding or compartmentalizing excessive Na^+^ that has accumulated in the protoplast [3, 6]. Many studies have documented several genes involved in Na^+^ exclusion, such as salt overly sensitive (SOS) pathway genes (*SOS1*, *SOS2* and *SOS3*), as well as *Nax1* and *Nax2*, which extrude Na^+^ to extracellular spaces [7–10]. Another efficient manner to bypass ionic damage is the sequestration of excessive cytosolic Na^+^ by the transfer of monovalent cations to the vacuoles via NHX antiporters, which exchange Na^+^ with H^+^ [11].

The early symptom of plants suffering from water stress caused by high soil salinity is suppressed root water uptake and water flow [12]. It is universally recognized that plasma membrane intrinsic proteins (PIPs) and tonoplast intrinsic proteins (TIPs) play key roles in the mediation of water uptake and intercellular water flow [12]. Comparative transcriptomic analyses have shown that, generally, the downregulated expression of PIPs in roots is believed to prevent water loss arising from transpiration and water desorption to salinized soils [12]. However, TIPs constitute important machinery that responds to water stress and maintains cellular hydraulic potential by allowing water to rapidly flow between vacuoles and the cytoplasm, yet the roles of these proteins need to be extensively investigated [12].

Under salt stress conditions, plant hormone signaling pathways enable plants to respond in a timely manner and modulate metabolic changes under unfavorable environmental conditions. Abscisic acid (ABA) is an essential endogenous regulator that mediates plant cell water balance. Under salinity, ABA biosynthesis genes are rapidly expressed. The resulting induced ABA promotes stomatal closure and causes the accumulation of osmo-protectants to rectify osmotic conditions [13]. Similarly, jasmonic acid (JA) is induced by salinity and serves as an effective endogenous protectant against salt stress, conferring salinity tolerance to susceptible plants [14]. A previous study also revealed that salicylic acid (SA) signaling pathways are correlated with salt stress tolerance. SA mitigates both oxidative damage and reduced water loss during salt stress [15].

In natural saline landscapes, salt stresses imposed on plants are never uniform because the salinity gradients in most soils are spatially inconsistent [16]. The physiological responses of plants to homogenous salinity can explain root water uptake patterns only in saline soils, leaving the question of how plants are physiologically adapted to variable salinity distributions around their root zones unanswered [17]. To answer this question, a split-root system was adopted to simulate the spatially nonuniform salinity conditions in real saline environments. Researchers using split-root systems have usually divided the roots of plants into two or more equal portions and have irrigated them with NaCl solutions of various concentrations, showing that, compared with that on plants exposed to a uniform salt treatment, salt toxicity on plants exposed to nonuniform salinity is mitigated along with improved plant performance because of more water and nutrients and less Na^+^ uptake from the lower-salinity root portion than from the higher-salinity portion [16, 18–20]. However, additional detailed information on the improved growth resulting from nonuniform salt stress has not yet been given.

The present study aimed to investigate the physiological alterations and the short-term transcriptomic responses to nonuniform salt stress conditions via a split-root system, in attempt to explain the molecular mechanisms underlying the mitigated growth of plants subjected to unequal concentrations of NaCl. Major parameters investigated in the present study included (1) status changes of physiological parameters such as photosynthesis and stress tolerance-related enzyme activity and (2) short-term expression patterns of stress-induced genes that counteract salinity-induced damage in a split-root system involving nonuniform salt stress.

## Material and methods

### Plant materials and salinity treatment

Jiliang 1, a sorghum variety developed at the Crop Research Institute, Shandong Academy of Agricultural Sciences, was used for this study. Plump seeds of uniform size were sown in sterilized wet sand in a nursery tray and germinated in a growth chamber at 25°C for 2 days. The root tips of germinated seeds were removed to stimulate lateral root growth. At the trefoil stage, the seedlings were subjected to a split-root system as described by Kong et al. [6], with modifications. The roots of each seedling were evenly divided in half, with each half placed in one of the two wells of a tray such that roots of the same seedling could be subjected to different salt stresses simultaneously. The seedlings in the split-root system were hydroponically cultivated in Hoagland solution for 2 weeks, and seedlings with healthy roots were retained. Nonuniform salt stress was then administered to the seedlings by exposing the two root portions to unequal concentrations of NaCl in Hoagland solution. The uniform salinity treatment (100/100 mmol·L^-1^ NaCl) was applied to both root portions in the two separate wells. However, for the nonuniform salinity treatment (0/200 mmol·L^-1^ NaCl), NaCl-free conditions (0 mmol·L^-1^) were implemented in one well, whereas salt stress (200 mmol·L^-1^) was imposed in the other well. Both root portions of the seedlings under NaCl-free conditions (0/0 mmol·L^-1^) were denoted as controls. Experimental samples prepared in the same batch were used for physiological status assessments and whole-genome expression profiling analyses.

### Experimental material sampling and statistical analysis

The leaves and roots of three different seedlings with similar appearances in each salinity treatment were sampled to determine changes in physiological status. For RNA-seq analysis, each treatment involved nine different seedlings and was replicated three times. Leaf samples from each replicate in each treatment were combined together for total RNA extraction. Root samples were collected according to the same sampling protocol as that used for the leaves. The significance levels of the different salinity treatments were defined using analysis of variance (ANOVA) via Minitab 17.3.1 (Minitab Inc., State College, PA, USA).

### Measurement of physiological indicators

The leaves and roots of plants from the cultivation trays were sampled for physiological measurements 6 hours after the stress was imposed. Dry weights were recorded after the samples were oven-dried at 130°C for 24 hours at 2 weeks after the stress was imposed. The photosynthesis and transpiration rates and the stomatal conductance were measured for the top three fully expanded leaves with a portable photosynthesis system (LI-COR Biosciences, USA), and the chlorophyll content of the third young fully expanded leaf was measured with a chlorophyll meter (TOP Cloud-agri, Zhejiang).

### Assessment of antioxidative enzyme activities

The antioxidative enzymes assayed in the study included peroxidase (POD), superoxide dismutase (SOD), and catalase (CAT). To assess the activities of these enzymes, 0.5 g of fresh leaf samples was homogenized in 100 mmol·L^-1^ sodium phosphate buffer (pH 7.0). The homogenates were subsequently centrifuged at 9000 × g at 4°C for 5 min. Thereafter, the supernatants were retained for the enzymatic assays. The POD activity was measured via the method reported by Klapheck et al. [21]. The nitro blue tetrazolium (NBT) method was to assay the SOD activity according to the methods of Giannopolitis and Ries [22]. Last, the method detailed by Aebi [23] was used to assess the CAT activity.

### Determination of the contents of salt stress-responsive nonenzymatic compounds

The contents of phytochemicals, proline (Pro) and reduced glutathione (GSH), which accumulate in leaves when plants are exposed to salinity, were determined. The GSH contents in the samples treated with different salinity levels were measured according to the same methods used by Bagheri et al. [24], and the Pro contents were estimated according to the method proposed by Bates et al. [25].

### Measurement of ion and nutrient accumulations in the leaves and roots

The Na^+^ and K^+^ concentrations in the leaves and roots were measured with a flame spectrophotometer (INSEA Analytical Instrument Co., Ltd., Shanghai) according to the method reported by Xiong et al. [26]. In addition, in accordance with the methods of Huang et al. [27], nitrate concentrations in the roots were measured for plants subjected to both uniform and nonuniform treatments.

### RNA extraction, cDNA library construction and sequencing

The physiological changes of the sorghum seedlings were analyzed, and the seedlings were subjected to RNA-seq analysis. Leaf and root samples were rapidly collected at 6 hours after the stress was imposed for RNA extraction. Total RNA was obtained and purified via the TRIzol reagent (BioFlux, Hangzhou) extraction method according to the manufacturer’s instructions, with some modifications as described by Zhang et al. [28]. RNA quality and quantity were determined for a small fraction of the total RNA extracts with a NanoDrop ND-1000 spectrophotometer (Nanodrop, Wilmington, DE, USA). The quantity of total RNA extracts was determined with a Qubit 2.0 fluorometer (Life Technologies, Carlsbad, USA), and the integrity was confirmed by electrophoresis on a 1% agarose gel. High-quality total RNA (100 ng·mL^-1^) from each stress treatment and the control group was used for cDNA library construction in accordance with the standard sample preparation protocol for the Illumina HiSeq^TM^ 2500 platform according to Liu et al. [29]. Sequencing of the constructed cDNA libraries was carried out by Biomarker Technology Co., Ltd. (Beijing, China).

### RNA-seq read mapping and transcript assembly

The total RNA raw reads generated from RNA-seq were filtered to remove any adaptors, empty reads and low-quality sequences that had more than 50% bases and that had a Q-value less than 20 as well as those sequences containing more than 10% missing bases prior to assembly [30]. This procedure resulted in high-quality clean reads that were mapped to the *Sorghum bicolor* reference genome (Sorghum_bicolor_NCBIv3) [31] to identify the sequencing reads with TopHat [32]. A reference annotation-based transcript assembly was subsequently established via the aligned reads in Cufflinks [33].

### Differentially expressed gene (DEG) identification and functional annotation

To identify DEGs, the DESeq2 algorithm was used to filter all unigenes across three leaf samples and four root samples [34]. The gene expression levels among the control and experimental groups of uniform salinity and nonuniform salinity were normalized based on the reads per kilobase per million reads (RPKM) values. The cutoff criteria for screening DEGs included a false discovery rate (FDR) ≤ 0.01 and a fold change (FC) ≥ 2.0 [29]. Functional annotation of the reads uniquely mapped to gene regions of the reference genome was performed by BLAST2GO to align the reads with those in the Gene Ontology (GO, https://www.geneontology.org) and Swiss-Prot databases (ftp://ftp.ebi.ac.uk).

### Gene expression validation

To confirm the gene expression profiles of the leaf and the root samples in both the uniform and nonuniform salinity treatments, the DEGs were randomly selected for quantitative real-time PCR (q-PCR) amplification. The same RNA samples used for RNA-seq were used for the determination of gene transcript levels. A q-PCR assay was performed according to described previously methods [35]. Primers for the selected genes and the internal control gene actin were designed with Primer Premier 6.24 (Premier Biosoft International, Palo Alto, CA, USA) and synthesized via Invitrogen Superscript II (Burlington, ON, Canada) at Biomarker Technology Co., Ltd. (Beijing, China); detailed information is listed in **S1 Table**. The expression levels of the selected genes were normalized to the expression level of actin and compared to the levels in the leaves and roots of plants in the nonstressed control group. The q-PCR results were correlated with the RNA-seq results via Minitab 17.3.1 (Minitab Inc., PA, USA).

## Results

### Estimation of physiological status in the uniform and nonuniform salinity treatments

**Fig 1:**
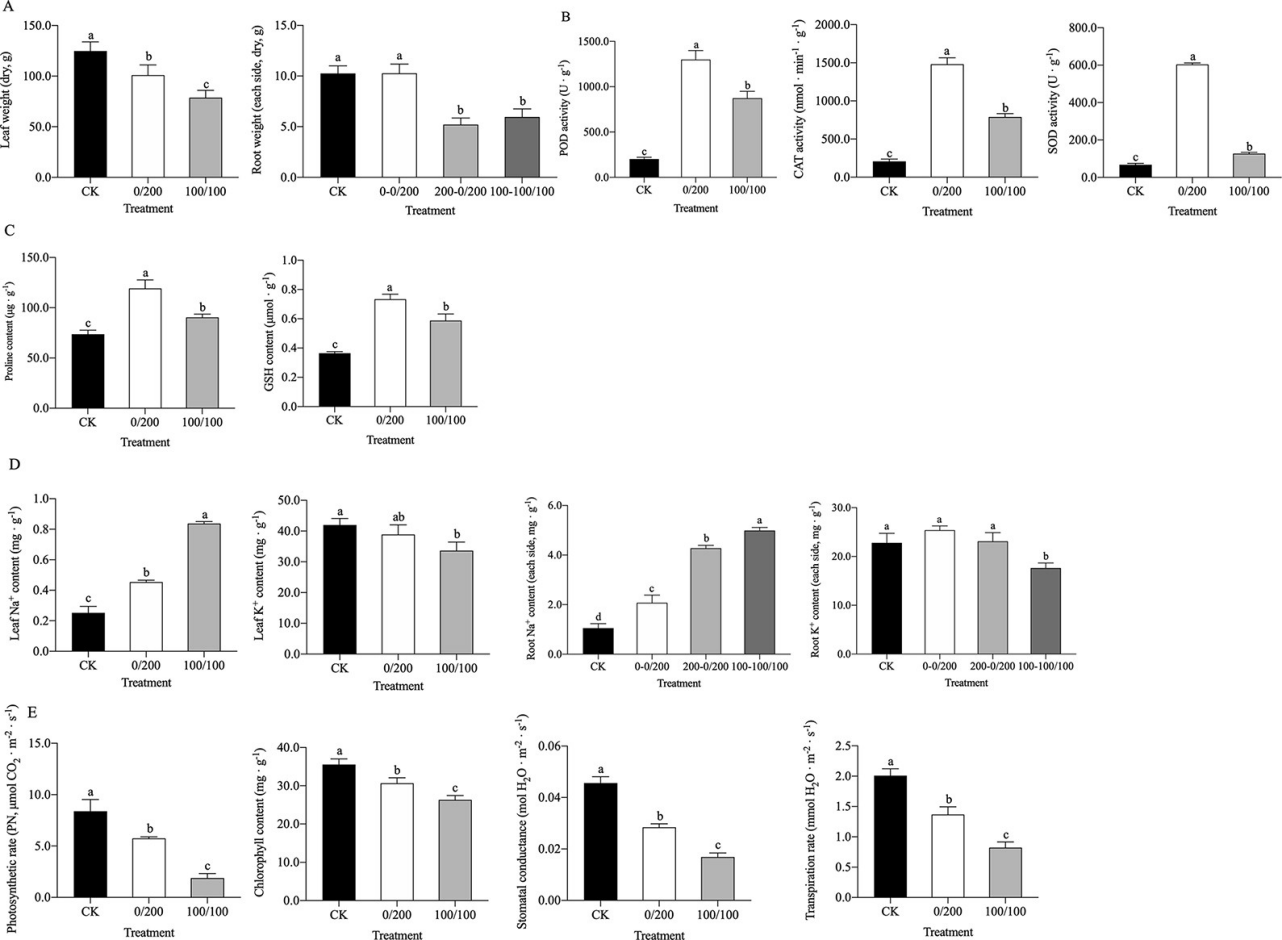
Effects of nonuniform (0 mmol·L^-1^ and 200 mmol·L^-1^ NaCl) and uniform (100 mmol·L^-1^ and 100 mmol·L^-1^ NaCl) salinity distribution on the dry weights of leaves and roots (**A**, measured two weeks after stress), activities of antioxidative enzymes (**B**), contents of Pro and GSH (**C**), Na^+^ and K^+^ distribution within the different leaf and root sides (**D**, 0–0/200 represents the nonsaline root side, 200–0/200 represents the high-saline root side, and 100–100/100 represents the uniform salinity treatment), and photosynthetic parameters (**E**). The bars represent the mean values of the investigated physiological indexes. The whiskers represent the standard deviations of the investigated physiological indexes. The treatments sharing the same letters do not significantly differ.

We measured the fresh weight, antioxidative enzyme activities, osmolyte concentrations, ionic accumulation and photosynthetic parameters of sorghum seedlings in both salinity treatments. Our results showed that nonuniform salinity (0/200 mmol·L^-1^ NaCl) could alleviate the impaired growth pattern of seedlings observed for those in the uniform salinity treatment (100/100 mmol·L^-1^ NaCl), considering that the total accumulated dry mass (measured two weeks after stress) was significantly greater (*P* ≤ 0.05) than that of their counterparts (**Fig 1A**). Likewise, the enzymatic activities of CAT, POD, and SOD in the leaves were largely reduced in the uniform salinity group compared with the nonuniform salinity group (*P* ≤ 0.05) (**Fig 1B**), and the accumulation of Pro and GSH increased in the nonuniform salinity group (**Fig 1C**). In the leaves and roots, ionic injury was greater in the uniform salinity group because of the greater Na^+^ and lower K^+^ contents present in the uniform salinity group compared with the other group (**Fig 1D**). Notably, the performance of key factors (**Fig 1E**) affecting photosynthesis was restricted further in the uniform salinity group than in the nonuniform salinity group at six hours after stress imposition.

### Statistical analysis of the RNA-seq data

To investigate the changes in gene expression at 6 hours after stress at the transcriptional level, the RNA-seq results were analyzed separately for the sorghum leaves and roots in the uniform and nonuniform salinity treatments. After a quality check was performed and null reads were excluded, 75.85 Gb (Q30 ≥ 90.25%) and 87.84 Gb (Q30 ≥ 89.77%) of paired-end clean data were generated in total for the leaf and root samples, respectively. In each library, the paired-end clean reads ranged from 24.43 Mb to 31.29 Mb and from 22.82 Mb to 26.27 Mb for the leaf and root samples, respectively. The genome of *Sorghum bicolor* was used as a reference genome for the alignment and gene mapping. Among the aligned reads, 82.35%–83.10% were mapped to the reference genome, and 81.04% of the mapped reads were unique to the leaf samples. On the other hand, mapped reads in the root samples accounted for 76.31%–80.31% of the total clean reads, and 74.84%–78.67% of the mapped reads were uniquely mapped to genic regions (**S2 Table**). The RNA-seq data were submitted to the Sequence Read Archive (SRA) of the NCBI database (submission number: **SUB4935186**).

### Identification of DEGs

**Fig 2:**
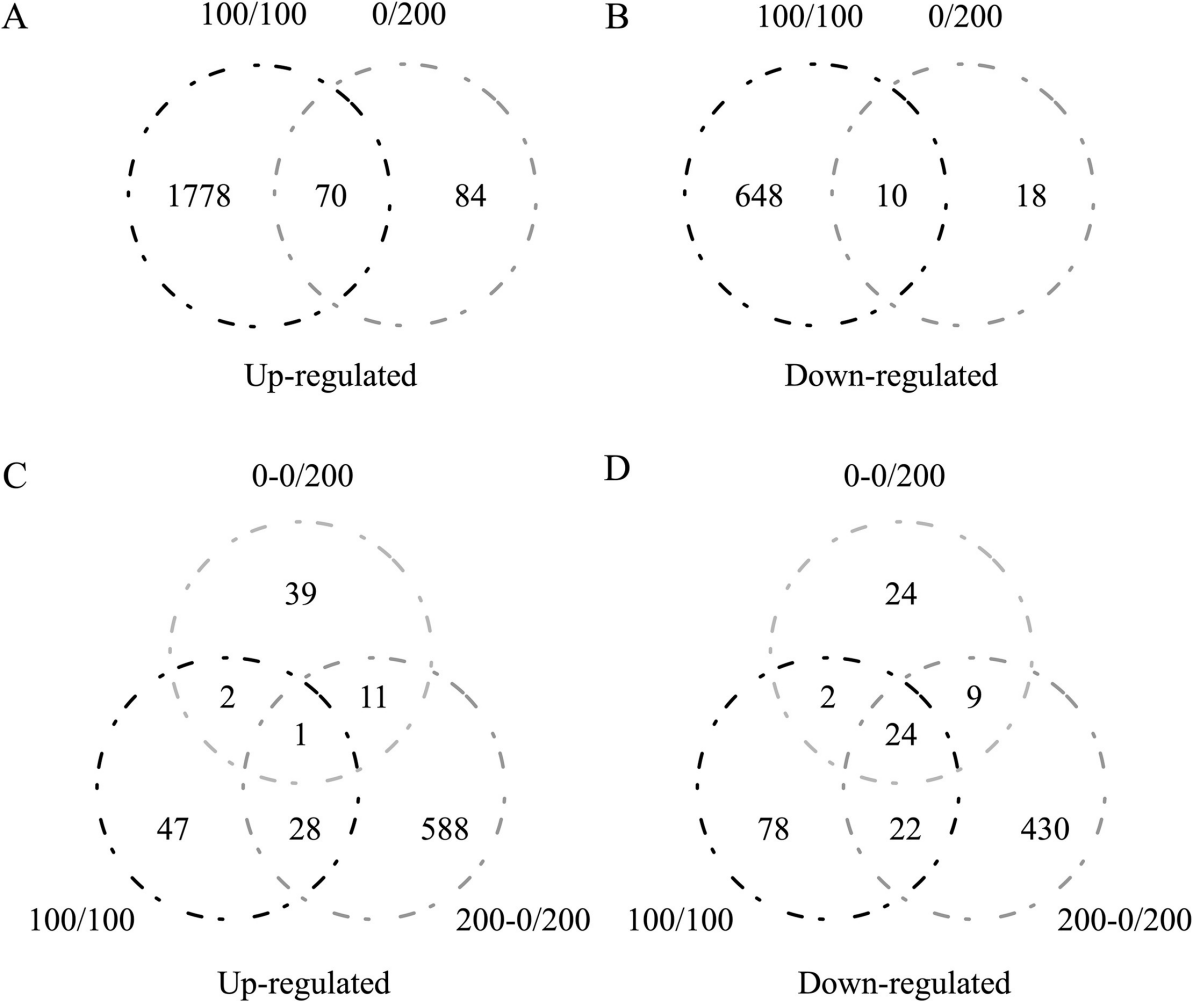
Venn diagram of DEGs whose expression was upregulated (**A**) or downregulated (**B**) in the leaves and upregulated (**C**) and downregulated (**D**) in the roots in the uniform (100 mmol·L^-1^ NaCl) and nonuniform (0 mmol·L^-1^ and 200 mmol·L^-1^ NaCl) salinity treatments, as revealed by RNA-seq.

Given the expression profiles from the RNA-seq data, DEGs were determined if the fold change in their expression was ≥ 2 and if their FDR value was ≤ 0.01. Accordingly, in the leaf samples, the expression of 1932 genes was upregulated under the uniform and nonuniform salinity stresses (**Fig 2A**), whereas 668 downregulated genes were identified as being expressed in the salinity treatment group compared with the control group (**Fig 2B**). With respect to the root samples, the expression of 716 genes was upregulated (**Fig 2C**), and the expression of 589 genes was downregulated (**Fig 2D**) in the uniform and nonuniform salinity treatments, respectively.

### Functional analysis of DEGs in the leaves

**Fig 3:**
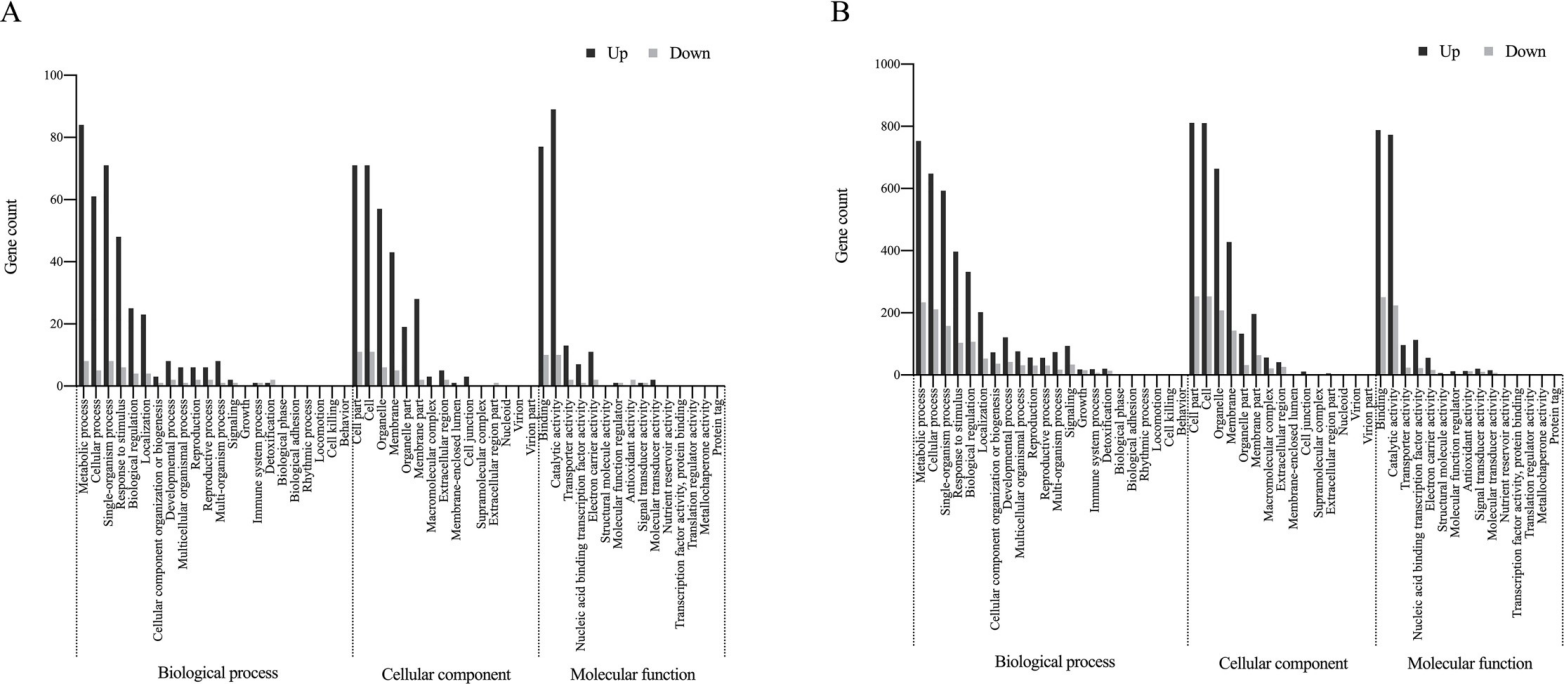
GO analysis of DEGs in the leaves in the nonuniform salinity treatment (0/200) (**A**) and uniform salinity treatment (100/100) (**B**), as determined by RNA-seq. The abscissa of the bar plot represents the gene count within each GO category. All processes listed presented enrichment when *P* < 0.01.

Functional annotation of the DEGs was conducted by mapping the sequences to the functional records in the GO database. **Fig 3** shows the GO annotation results of the DEGs in the leaves under nonuniform and uniform salinity conditions. Among the DEGs, functional analysis revealed six genes related to salt/osmotic stress responses; a group of genes coding for antioxidants; 12 determinant genes whose products regulate the syntheses of phytohormones such as auxin, ethylene, JA and SA; seven genes encoding salt stress-associated transcription factors (TFs); a number of Na^+^ and K^+^ transport-related genes; salt compartmentalizing pathway-related genes; and genes encoding ATP-binding cassette (ABC) transporters, along with several genes involved in photosynthesis and stomatal movement. The expression of these genes was upregulated in the nonuniform salinity treatment but remained unchanged in the uniform salinity treatment (**Table 1**).

**Table 1:** Summary of differentially expressed genes in leaves up-regulated under non-uniform salinity condition.

Functional category	Locus name	100–100^a^	0–200^a^	Gene annotation
Response to salt stress	LOC8062357	-0.48	3.02	Chitinase (*Hevea brasiliensis*)
Response to oxidative stress	LOC8058481	-0.10	7.39	Peroxidase 54 (Precursor) (*Arabidopsis thaliana*)
	LOC8058486	-0.11	4.15	Peroxidase 54 (Precursor) (*Arabidopsis thaliana*)
	LOC8056546	-0.41	3.46	Peroxidase 25 (Precursor) (*Arabidopsis thaliana*)
	LOC8068221	-0.95	2.66	Catalase 3 (Precursor) (*Arabidopsis thaliana*)
	LOC8069231	-0.57	2.10	Catalase 1 (Precursor) (*Arabidopsis thaliana*)
	LOC110431820	-1.53	1.58	Superoxide dismutase 4 (Precursor) (*Arabidopsis thaliana*)
Response to osmotic stress	LOC8060121	-0.05	1.83	RNA polymerase II C-terminal domain phosphatase-like 2 (*Arabidopsis thaliana*)
	LOC8060260	-0.05	1.50	Phospholipase D alpha 1 (*Vigna unguiculata*)
	LOC110430077	-0.51	1.19	Annexin D4 (*Arabidopsis thaliana*)
ABA biosynthesis	LOC8062208	1.94	3.62	9-cis-epoxycarotenoid dioxygenase 1(*Zea mays*)
	LOC8084125	0.02	1.57	Indole-3-acetaldehyde oxidase (*Zea mays*)
	LOC8061592	0.06	1.21	Molybdenum cofactor sulfurase (*Oryza sativa* subsp. *japonica*)
Auxin biosynthesis	LOC8084125	0.02	1.57	Indole-3-acetaldehyde oxidase (Zea mays)
	LOC110436279	0.83	1.26	Anthranilate synthase beta subunit 1 (*Oryza sativa* subsp. *japonica*)
Jasmonic acid biosynthesis	LOC8070774	0.77	6.40	12-oxophytodienoate reductase 1 (*Oryza sativa* subsp. *japonica*)
	LOC8070775	1.15	5.98	12-oxophytodienoate reductase 1 (*Oryza sativa* subsp. *japonica*)
	LOC8070776	0.83	5.95	12-oxophytodienoate reductase 1 (*Oryza sativa* subsp. *japonica*)
Salicylic acid biosynthesis	LOC110431248	0.25	2.37	Mitogen-activated protein kinase 5 (*Oryza sativa* subsp. *japonica*)
	LOC8072566	0.59	1.56	Ammonium transporter 2 member 1 (*Oryza sativa* subsp. *japonica*)
	LOC8060585	0.22	1.54	Chitin elicitor receptor kinase 1 (Precursor) (*Oryza sativa* subsp. *japonica*)
Photosynthesis	LOC8062358	-0.02	2.87	Ferredoxin-6 (*Zea mays*)
	LOC110431248	-0.25	2.37	Mitogen-activated protein kinase 5 (*Oryza sativa* subsp. *japonica*)
	LOC8081748	-0.80	1.29	Chlorophyll a-b binding protein of LHCII type III (*Hordeum vulgare*)
Transcriptional factors	LOC8057074	0.09	3.87	NAC domain-containing protein 67 (*Oryza sativa* subsp. *japonica*)
	LOC8067417	-0.01	2.99	Myb-related protein Myb4 (*Oryza sativa* subsp. *japonica*)
	LOC8058421	-0.20	6.24	Probable WRKY transcription factor 40 (*Arabidopsis thaliana*)
	LOC8076973	0.06	5.69	Ethylene-responsive transcription factor ERF020 (*Arabidopsis thaliana*)
	LOC8080477	0.20	4.62	Zinc finger protein 2 (*Arabidopsis thaliana*)
	LOC8061169	-0.30	2.13	Light-inducible protein CPRF2 (*Petroselinum crispum*)
	LOC8055721	1.13	4.04	Putative transcription factor bHLH041 (*Arabidopsis thaliana*)
	LOC8055146	-0.15	3.57	PLATZ transcriptional factor (*Arabidopsis thaliana*)
Proline	LOC8074727	0.72	4.51	Probable polyamine oxidase 2 (*Arabidopsis thaliana*)
	LOC8057441	0.77	3.83	Rhodanese-like domain-containing protein 6 (*Arabidopsis thaliana*)
Glutathione	LOC8055934	0.57	3.23	Glutamate dehydrogenase 2 (*Arabidopsis thaliana*)
	LOC8063168	-0.18	3.30	Probable glutathione S-transferase GSTU6 (*Oryza sativa subsp*. *japonica*)
	LOC8081335	-0.02	3.01	Probable glutathione S-transferase (*Nicotiana tabacum*)
	LOC8083917	0.55	2.97	Probable glutathione S-transferase GSTU1 (*Oryza sativa* subsp. *japonica*)
Potassium transporter	LOC8078559	0.30	2.40	Probable potassium transporter 9 (*Oryza sativa* subsp. *japonica*)
	LOC8072046	0.54	1.85	Potassium transporter 1 (*Oryza sativa* subsp. *japonica*)
	LOC8074423	0.23	1.16	Potassium transporter 7 (*Oryza sativa* subsp. *japonica*)
	LOC8058236	0.48	3.87	Potassium channel AKT1 (*Oryza sativa* subsp. *japonica*)
Sodium related transporter	LOC8064842	-0.04	2.21	Tonoplast dicarboxylate transporter (*Arabidopsis thaliana*)
	LOC8071496	0.70	2.05	Sodium/calcium exchanger 1 (*Arabidopsis thaliana*)
	LOC8055193	0.91	2.00	Tonoplast dicarboxylate transporter (*Arabidopsis thaliana*)
	LOC8057705	0.23	1.59	Glutamate receptor 3.1 (Precursor) (*Oryza sativa* subsp. *japonica*)
	LOC8081867	0.30	1.52	Calcium-transporting ATPase 1 (*Oryza sativa* subsp. *japonica*)
	LOC8055864	0.74	1.46	Tonoplast dicarboxylate transporter (*Arabidopsis thaliana*)
	LOC8085208	0.70	1.39	Glutamate receptor 3.1 (Precursor) (*Oryza sativa* subsp. *japonica*)
	LOC8073506	0.19	1.24	Putative phospholipid-transporting ATPase 8 (*Arabidopsis thaliana*)
	LOC8083686	0.25	1.23	Phospholipid-transporting ATPase 1 (*Arabidopsis thaliana*)
	LOC8061446	0.26	1.09	K^+^ efflux antiporter 5 (*Arabidopsis thaliana*)
	LOC8064926	0.15	1.00	Phospholipid-transporting ATPase 3 (*Arabidopsis thaliana*)
CBL-interacting	LOC8060301	-0.85	3.29	CBL-interacting protein kinase 1 (*Oryza sativa* subsp. *japonica*)
serine/thieonine protin	LOC8060254	1.28	2.08	CBL-interacting protein kinase 16 (*Oryza sativa* subsp. *japonica*)
kinase	LOC8077845	1.40	4.38	CBL-interacting protein kinase 6 (*Oryza sativa* subsp. *japonica*)
	LOC8085707	0.81	1.93	CBL-interacting protein kinase 9 (*Oryza sativa* subsp. *japonica*)
	LOC8060255	0.80	1.12	Putative CBL-interacting protein kinase 27 (*Oryza sativa* subsp. *japonica*)
ABC transporter	LOC8083267	0.17	2.78	ABC transporter G family member 11 (*Arabidopsis thaliana*)
	LOC8060300	0.23	1.86	ABC transporter B family member 21 (*Arabidopsis thaliana*)
	LOC8078474	0.16	1.73	ABC transporter C family member 3 (*Arabidopsis thaliana*)
	LOC8079723	0.53	1.35	ABC transporter G family member 25 (*Arabidopsis thaliana*)
	LOC8069652	0.24	1.17	ABC transporter G family member 14 (*Arabidopsis thaliana*)
	LOC8080533	0.54	1.10	ABC transporter G family member 22 (*Arabidopsis thaliana*)
	LOC8086346	0.23	1.03	ABC transporter C family member 14 (*Arabidopsis thaliana*)
	LOC8076723	1.04	0.47	ABC transporter C family member 8 (Precursor) (*Arabidopsis thaliana*)

a, represents fold changes calculated from log_2_ of RPKM values of experimental samples (100/100, uniform salinity treatment; 0/200, non-uniform salinity treatments, respectively) vs those of CK.

### Functional analysis of DEGs in the roots

**Fig 4:**
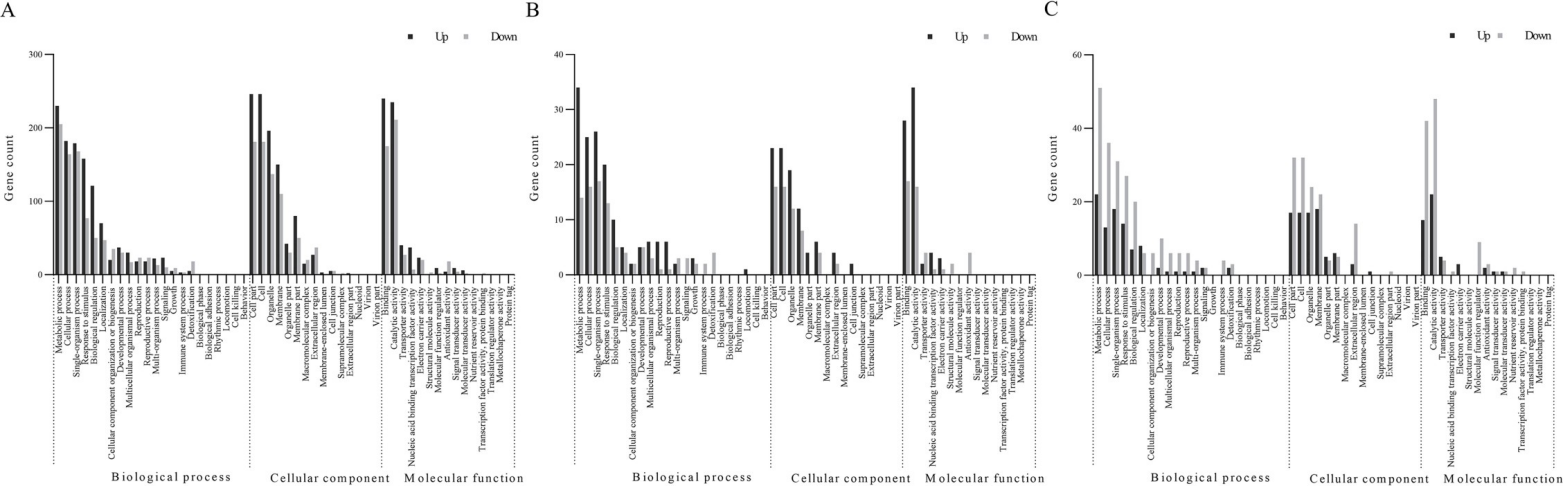
GO analysis of DEGs in the roots of the high-saline root side (200–0/200) (**A**), of the nonsaline root side (0–0/200) (**B**) and in the uniform salinity treatment (100–100/100) (**C**), as determined by RNA-seq. The abscissa of the bar plot represents the gene count within each GO category. All processes listed presented enrichment when *P* < 0.01.

GO analysis revealed the GO terms that were assigned to the root sides in the uniform salinity treatment (100 mmol·L^-1^ and 100 mmol·L^-1^ NaCl) and the saline (200 mmol·L^-1^ NaCl) and the nonsaline (0 mmol·L^-1^ NaCl) root sides in the nonuniform salinity treatment, as presented in **Fig 4**. The major DEGs of interest were genes involved in the ABA synthesis pathway and water and mineral uptake, as well as those encoding TFs that respond to salt stress. Notably, the expression of two 9-cis-epoxycarotenoid dioxygenase 1 genes, which are essential for ABA biosynthesis, was upregulated in the high-saline side in the nonuniform salinity treatment; however, the expression was normal both in the nonsaline side in the nonuniform salinity treatment and in the uniform salinity treatment. Furthermore, genes repressing ABA biosynthesis were expressed at relatively high levels in the NaCl-free side and in the uniformly stressed seedlings. Moreover, the expression of genes involved ABA-mediated signaling pathways was upregulated on the high-saline root side (**Table 2**). We identified a total of 22 DEGs coding for aquaporins, PIPs and TIPs. In terms of their expression, most PIP genes were largely inhibited in the salinity treatment compared with the nonsaline side in the nonuniform salinity treatment, whereas the expression of most of the TIPs increased in the nonsaline root side of the nonuniform salinity treatment compared with the other treatment (**Table 3**). Similarly, the expression of genes encoding transporters of K^+^, nitrate, and phosphate was downregulated in both salinity treatments, whereas compared with that in both the high-saline side and the uniform salinity treatment, the expression in the NaCl-free side of the nonuniform salinity treatment was upregulated (**Table 4**). In response to salt stress, the expression levels of a number of TFs, for example, NACs, ERFs, WRKYs, MYBs, and bZIPs, changed. Specifically, in the high-saline side in the nonuniform salinity treatment, the expression of most of the genes encoding salt stress-responsive TFs was upregulated, while the expression levels of the DEGs encoding TFs did not differ in the nonsaline root side in the nonuniform salinity treatment and in the uniform counterpart (**Table 5**).

**Table 2:** Summary of differentially expressed ABA related genes in roots under uniform and non-uniform salinity treatments.

Locus name	100/100^a^	0-0/200^a^	200-0/200^a^	Gene annotation
LOC8081132	1.32	0.33	3.99	9-cis-epoxycarotenoid dioxygenase 1, chloroplastic (Precursor) (*Zea mays*)
LOC8084948	1.26	0.04	3.23	Probable protein phosphatase 2C 30 (*Oryza sativa* subsp. *japonica*)
LOC110430337	0.59	-0.43	3.01	Probable protein phosphatase 2C 49 (*Oryza sativa* subsp. *japonica*)
LOC8075201	0.90	-0.35	3.01	Abscisic acid-insensitive 5-like protein 6 (*Arabidopsis thaliana*)
LOC8080659	0.82	0.30	2.72	Protein HVA22 (*Hordeum vulgare*)
LOC8075822	0.77	0.11	2.51	Protein HVA22 (*Hordeum vulgare*)
LOC8062208	0.59	0.30	2.40	9-cis-epoxycarotenoid dioxygenase 1, chloroplastic (Precursor) (*Zea mays*)
LOC8061070	0.53	-0.23	2.32	Probable protein phosphatase 2C 50 (*Oryza sativa* subsp. *japonica*)
LOC8054565	1.08	-0.41	2.16	Probable protein phosphatase 2C 68 (*Oryza sativa* subsp. *japonica*)
LOC110433516	1.24	-0.20	2.14	Gamma-glutamyl phosphate reductase (*Oryza sativa* subsp. *japonica*)
LOC8073213	1.21	-0.03	2.13	Unknown domain
LOC8058897	0.96	-0.01	2.09	Probable protein phosphatase 2C 9(*Oryza sativa* subsp. *japonica*)
LOC8056555	0.34	-0.35	1.95	Galactinol-sucrose galactosyltransferase (*Oryza sativa* subsp. *japonica*)
LOC8062479	1.29	-0.12	1.88	Type I inositol 1,4,5-trisphosphate 5-phosphatase 11 (*Arabidopsis thaliana*)
LOC8055473	0.30	-0.35	1.86	NAC domain-containing protein 7 (*Arabidopsis thaliana*)
LOC8077354	0.09	0.28	1.83	bZIP transcription factor TRAB1 (*Oryza sativa* subsp. *japonica*)
LOC8078643	0.92	0.15	1.77	Probable protein phosphatase 2C 6 (*Oryza sativa* subsp. *japonica*)
LOC8082252	0.71	0.31	1.77	Transcription factor HY5 (*Arabidopsis thaliana*)
LOC110436123	0.41	-0.07	1.71	Zeaxanthin epoxidase, chloroplastic (Precursor) (*Oryza sativa* subsp. *japonica*)
LOC8080995	0.42	0.04	1.69	Aldehyde dehydrogenase family 7 member A1 (*Malus domestica*)
LOC8054855	0.73	0.26	1.66	UDP-glycosyltransferase 74F2 (*Arabidopsis thaliana*)
LOC8075332	0.40	-0.21	1.62	Abscisic stress-ripening protein 2 (*Solanum lycopersicum*)
LOC110434011	0.58	0.29	1.60	Protein MAO HUZI 4 (*Sorghum bicolor*)
LOC8072538	0.80	-0.07	1.56	Unknown domain
LOC110429605	0.62	0.21	1.55	Abscisic stress-ripening protein 2 (*Solanum lycopersicum*)
LOC8077039	0.20	0.07	1.52	Cysteine-rich receptor-like protein kinase 25 (Precursor) (*Arabidopsis thaliana*)
LOC8079723	0.82	0.81	1.47	ABC transporter G family member 25 (*Arabidopsis thaliana*)
LOC8081161	0.25	-0.16	1.46	Indole-3-acetaldehyde oxidase (*Zea mays*)
LOC8072010	0.37	-0.04	1.40	Protein TIFY 9 (*Arabidopsis thaliana*)
LOC8076977	0.22	-0.13	1.39	NAC domain-containing protein 48 (*Oryza sativa subsp*. *japonica*)
LOC8065807	0.38	-0.59	1.36	LanC-like protein GCR2 (*Arabidopsis thaliana*)
LOC8077115	0.20	-0.21	1.29	Serine/threonine-protein kinase SAPK1 (*Oryza sativa* subsp. *japonica*)
LOC8054342	0.20	0.19	1.18	ACT domain-containing protein ACR8 (*Arabidopsis thaliana*)
LOC8071261	0.17	0.15	1.18	Phosphoinositide phospholipase C2 (*Arabidopsis thaliana*)
LOC110437038	0.18	-0.25	1.08	bZIP transcription factor TRAB1 (*Oryza sativa* subsp. *japonica*)
LOC8085470	-0.07	-0.47	-2.62	Inorganic phosphate transporter 1–2 (*Oryza sativa* subsp. *japonica*)
LOC8081117	-1.02	0.04	-2.13	Abscisic acid receptor PYL4 (*Arabidopsis thaliana*)
LOC8061804	-1.10	-0.05	-1.77	Abscisic acid receptor PYL4 (*Arabidopsis thaliana*)
LOC8066580	0.20	0.15	-1.61	Abscisic acid 8’-hydroxylase 4 (*Arabidopsis thaliana*)
LOC8055017	-0.43	0.50	-1.56	Bidirectional sugar transporter SWEET14 (*Oryza sativa* subsp. *japonica*)
LOC8073793	-0.29	-0.25	-1.39	Abscisic acid receptor PYL2 (*Arabidopsis thaliana*)
LOC8074867	-0.55	-1.43	-1.30	CBL-interacting protein kinase 14 (*Oryza sativa* subsp. *japonica*)
LOC8076279	0.01	0.27	-1.24	Fructose-bisphosphate aldolase, chloroplastic (Precursor) (*Oryza sativa* subsp. *japonica*)
LOC8057655	-0.72	-1.04	-0.36	Tetraketide alpha-pyrone reductase 2 (*Arabidopsis thaliana*)

a, represents fold changes calculated from log_2_ of RPKM values of experimental samples (100/100, uniform salinity; 0-0/200 and 200-0/200, non-saline and high saline root sides of non-uniform salinity treatments, respectively) vs those of CK.

**Table 3:** Summary of differentially expressed aquaporins in roots under uniform and non-uniform salinity treatments.

Locus name	100/100^a^	0-0/200^a^	200-0/200^a^	Gene annotation
LOC8085362	-2.90	-1.57	-3.08	Probable aquaporin TIP2-2 (*Oryza sativa* subsp. *japonica*)
LOC8076232	-1.14	1.26	-1.19	Aquaporin TIP2-1 (*Zea mays*)
LOC8078014	-1.09	2.12	-1.13	Probable aquaporin TIP3-1 (*Oryza sativa* subsp. *japonica*)
LOC8055965	-1.16	1.24	-2.95	Aquaporin TIP2-3 (*Zea mays*)
LOC8064643	-1.24	1.08	-1.83	Aquaporin PIP1-2 (*Zea mays*)
LOC8066174	-1.19	1.14	-2.75	Aquaporin PIP2-4 (*Zea mays*)
LOC8155434	-2.06	2.31	-2.71	Aquaporin PIP2-3 (*Zea mays*)
LOC8055964	1.35	3.75	-1.50	Aquaporin TIP5-1 (*Zea mays*)
LOC8075940	1.10	2.44	-1.46	Aquaporin PIP2-5 (*Zea mays*)
LOC8080564	-2.37	-1.05	-2.44	Aquaporin TIP1-1 (*Zea mays*)
LOC8059750	-2.21	1.11	-2.43	Aquaporin PIP2-1 (*Zea mays*)
LOC8074142	1.01	2.30	-1.39	Probable aquaporin TIP1-2 (*Oryza sativa* subsp. *japonica*)
LOC8078102	-1.28	1.11	-1.34	Aquaporin TIP4-4 (*Zea mays*)
LOC8059751	1.55	1.51	-1.07	Aquaporin PIP2-6 (*Zea mays*)
LOC8057970	1.49	1.26	-1.06	Aquaporin PIP2-6 (*Zea mays*)
LOC8066640	-2.09	2.12	0.13	Aquaporin TIP4-2 (*Zea mays*)
LOC8059708	0.34	1.46	0.13	Aquaporin PIP1-5 (*Zea mays*)
LOC8076062	-1.04	1.14	-2.17	Aquaporin PIP1-2 (*Zea mays*)
LOC8057973	1.48	1.27	-1.21	Aquaporin PIP2-6 (*Zea mays*)
LOC8065486	1.23	1.23	-1.47	Aquaporin PIP1-6 (*Zea mays*)
LOC110432874	1.01	1.01	-1.11	Probable aquaporin PIP2-7 (*Oryza sativa* subsp. *japonica*)
LOC8082864	0.11	-1.03	1.89	Probable aquaporin TIP3-2 (*Oryza sativa* subsp. *japonica*)

a, represents fold changes calculated from log_2_ of RPKM values of experimental samples (100/100, uniform salinity; 0-0/200 and 200-0/200, non-saline and high saline root sides of non-uniform salinity treatments, respectively) vs those of CK.

**Table 4:** Summary of differentially expressed nitrate, potassium, and phosphate transporter genes in roots under uniform and non-uniform salinity treatments.

Nutrient	Locus name	100/100^a^	0-0/200^a^	200-0/200^a^	Gene annotation
Nitrate	LOC8061196	-0.40	1.56	-2.26	Probable peptide/nitrate transporter At3g43790 (*Arabidopsis thaliana*)
	NewGene_789	-1.29	0.39	-1.77	Protein NRT1/ PTR FAMILY 5.16 (*Arabidopsis thaliana*)
	LOC8066510	-1.04	0.38	-2.76	High-affinity nitrate transporter 2.2 (*Oryza sativa* subsp. *japonica*)
	LOC8054634	-0.25	1.42	-0.45	Probable peptide/nitrate transporter At3g43790 (*Arabidopsis thaliana*)
	LOC8069516	-0.46	0.65	-1.39	High-affinity nitrate transporter 2.3 (*Oryza sativa* subsp. *japonica*)
	LOC8070606	0.02	1.39	-0.30	High-affinity nitrate transporter-activating protein 2.1 (Precursor) (*Oryza sativa* subsp. *japonica*)
	LOC8060993	-0.08	-0.04	-2.96	Protein NRT1/ PTR FAMILY 5.10 (*Arabidopsis thaliana*)
	LOC8085192	-1.62	1.57	-2.49	High-affinity nitrate transporter 2.2 (*Oryza sativa* subsp. *japonica*)
	LOC8071422	-0.73	1.87	-1.51	Probable peptide/nitrate transporter At3g43790 (*Arabidopsis thaliana*)
	LOC8073590	0.12	0.40	0.14	High-affinity nitrate transporter-activating protein 2.1 (Precursor) (*Oryza sativa* subsp. *japonica*)
	LOC8085190	-0.38	0.27	-0.77	High-affinity nitrate transporter 2.2 (*Oryza sativa* subsp. *japonica*)
	LOC110435376	0.34	0.34	0.88	Protein NRT1/ PTR FAMILY 4.6 (*Arabidopsis thaliana*)
Potassium	LOC8085154	-1.11	1.36	-1.32	Putative potassium transporter 12 (*Oryza sativa* subsp. *japonica*)
	LOC8072341	-0.13	0.59	-0.39	Probable potassium transporter 11 (*Oryza sativa* subsp. *japonica*)
	LOC8056191	-0.20	-0.16	-0.31	Potassium transporter 23 (*Oryza sativa* subsp. *japonica*)
	LOC8061597	-1.41	0.19	-2.79	Probable potassium transporter 13 (*Oryza sativa* subsp. *japonica*)
	LOC8062281	-0.43	0.11	-1.84	Probable potassium transporter 2 (*Oryza sativa* subsp. *japonica*)
	LOC8074423	-0.14	0.20	-1.56	Potassium transporter 7 (*Oryza sativa* subsp. *japonica*)
	LOC8081576	0.17	1.64	0.42	Potassium transporter 22 (*Oryza sativa* subsp. *japonica*)
	LOC8072324	-0.46	-0.41	-1.74	Probable potassium transporter 15 (*Oryza sativa* subsp. *japonica*)
	LOC8072046	-0.51	-0.06	-1.92	Potassium transporter 1 (*Oryza sativa* subsp. *japonica*)
	LOC8082329	-0.05	0.01	0.30	Putative potassium transporter 8 (*Oryza sativa* subsp. *japonica*)
	LOC8061667	-1.07	0.35	-1.36	Potassium transporter 18 (*Oryza sativa* subsp. *japonica*)
	LOC8063025	0.02	0.91	0.43	Probable potassium transporter 16 (*Oryza sativa* subsp. *japonica*)
	LOC8067672	0.14	0.13	0.43	Potassium transporter 10 (*Oryza sativa* subsp. *japonica*)
	LOC8065633	-1.13	0.67	-1.44	Potassium transporter 21(*Oryza sativa* subsp. *japonica*)
	LOC8077489	0.01	0.07	0.60	Probable potassium transporter 14 (*Oryza sativa* subsp. *japonica*)
	LOC8078559	0.25	0.13	0.71	Probable potassium transporter 9 (*Oryza sativa* subsp. *japonica*)
	LOC8065785	-0.30	1.67	-1.72	Potassium transporter 25 (*Oryza sativa* subsp. *japonica*)
	LOC8062763	0.18	1.43	0.77	Probable potassium transporter 17 (*Oryza sativa* subsp. *japonica*)
	LOC8071732	-1.06	0.28	-1.32	Potassium transporter 25 (*Oryza sativa* subsp. *japonica*)
Phosphate	LOC8085470	-1.67	-0.47	-2.62	Inorganic phosphate transporter 1–2 (*Oryza sativa* subsp. *japonica*)
	LOC8070461	0.03	0.50	-1.74	Phosphate transporter PHO1-1 (*Oryza sativa* subsp. *japonica*)
	LOC8085471	0.12	0.33	-1.20	Probable inorganic phosphate transporter 1–12 (*Oryza sativa* subsp. *japonica*)
	LOC8078266	-0.96	1.21	-1.39	Probable inorganic phosphate transporter 1–10 (*Oryza sativa* subsp. *japonica*)
	LOC8069732	0.04	1.26	-0.39	Phosphate transporter PHO1-3 (*Oryza sativa* subsp. *japonica*)
	LOC8076626	-0.21	1.16	-0.83	Phosphate transporter PHO1-2 (*Oryza sativa* subsp. *japonica*)
	LOC8054833	0.41	0.65	-0.64	Inorganic phosphate transporter 1–6 (*Oryza sativa* subsp. *japonica*)
	LOC8071389	-0.40	0.27	-0.66	Probable inorganic phosphate transporter 1–8 (*Oryza sativa* subsp. *japonica*)
	LOC8056420	0.62	0.75	0.39	Phosphate transporter PHO1-2 (*Oryza sativa* subsp. *japonica*)
	LOC8055817	-0.89	0.12	-1.00	Inorganic phosphate transporter 2–1, chloroplastic (Precursor) (*Arabidopsis thaliana*)

a, represents fold changes calculated from log_2_ of RPKM values of experimental samples (100/100, uniform salinity; 0-0/200 and 200-0/200, non-saline and high saline root sides of non-uniform salinity treatments, respectively) vs those of CK.

**Table 5:** Summary of differentially expressed transcriptional factors in roots under uniform and non-uniform salinity treatments.

TFs	Locus name	100/100^a^	0-0/200^a^	200-0/200^a^	Gene annotation
NAC	LOC8069067	1.43	0.83	0.64	NAC domain-containing protein 43 (*Arabidopsis thaliana*)
	LOC8055613	-0.14	1.14	1.98	NAC domain-containing protein 45 (*Arabidopsis thaliana*)
	LOC8055473	0.30	-0.35	1.86	NAC domain-containing protein 7 (*Arabidopsis thaliana*)
	LOC8076977	0.22	-0.13	1.39	NAC domain-containing protein 48 (*Oryza sativa* subsp. *japonica*)
	LOC8084562	0.08	-0.27	1.21	NAC transcription factor NAM-B2 (*Triticum turgidum* subsp. *durum*)
	LOC8085025	-0.58	-0.90	-2.08	NAC domain-containing protein 21/22 (*Arabidopsis thaliana*)
ERF	LOC8082008	-1.02	-0.37	-1.16	Ethylene-responsive transcription factor ERF073 (*Arabidopsis thaliana*)
	LOC8073624	-0.05	-0.59	2.51	Ethylene-responsive transcription factor ERF114 (*Arabidopsis thaliana*)
	LOC110434463	0.04	0.27	1.60	Ethylene-responsive transcription factor ERF025 (*Arabidopsis thaliana*)
	LOC8083365	0.46	0.07	1.47	Ethylene-responsive transcription factor ERF034 (*Arabidopsis thaliana*)
	LOC8054804	-0.09	-0.11	1.78	Ethylene-responsive transcription factor ERF114 (*Arabidopsis thaliana*)
	LOC8081902	0.38	-0.07	1.14	Ethylene-responsive transcription factor RAP2-4 (*Arabidopsis thaliana*)
WRKY	LOC8074604	0.25	0.39	1.64	Probable WRKY transcription factor 71 (*Arabidopsis thaliana*)
	LOC8084860	0.60	0.29	1.62	Probable WRKY transcription factor 71 (*Arabidopsis thaliana*)
	LOC8071350	0.15	0.00	1.53	Probable WRKY transcription factor 30 (*Arabidopsis thaliana*)
	LOC8054521	0.62	0.42	1.21	Probable WRKY transcription factor 57 (*Arabidopsis thaliana*)
	LOC8057730	0.08	0.29	1.12	Probable WRKY transcription factor 33 (*Arabidopsis thaliana*)
	LOC110436263	-0.51	-0.15	-1.25	DNA-binding WRKY (*Zea mays*)
MYB	LOC8069000	1.77	0.95	1.28	Myb-related protein Hv33 (*Hordeum vulgare*)
	LOC8068849	1.57	-0.35	1.07	Transcription factor MYB108 (*Arabidopsis thaliana*)
	LOC8064455	1.24	-0.12	5.55	Myb-related protein 305 (*Antirrhinum majus*)
	LOC8086015	1.23	1.13	1.28	Myb-related protein MYBAS1 (*Oryza sativa* subsp. *japonica*)
	LOC8083751	1.23	0.39	0.53	Transcription factor MYB44 (*Arabidopsis thaliana*)
	LOC8056325	0.78	-0.37	2.71	Myb-related protein 308 (*Antirrhinum majus*)
	LOC8085503	0.10	-0.27	1.93	Transcription factor MYB57 (*Arabidopsis thaliana*)
	LOC8071730	-0.08	-0.03	1.80	Myb-related protein 305 (*Antirrhinum majus*)
	LOC8074528	0.35	0.10	1.73	Transcription factor MYB44 (*Arabidopsis thaliana*)
	LOC8054657	0.31	0.15	1.48	Transcription factor MYB44 (*Arabidopsis thaliana*)
	LOC8065632	0.40	0.28	1.35	Transcription factor GAMYB (*Oryza sativa* subsp. *indica*)
	LOC8083688	0.19	0.49	1.14	Myb family transcription factor APL (*Arabidopsis thaliana*)
	LOC8067939	0.10	0.25	1.10	Transcription factor RAX3 (*Arabidopsis thaliana*)
	LOC8059197	-0.88	-0.87	-1.87	Transcription factor RAX2 (*Arabidopsis thaliana*)
	LOC8078491	0.20	0.68	-1.71	Transcription factor MYB86 (*Arabidopsis thaliana*)
	LOC110434077	0.58	0.81	-1.65	Myb-related protein Hv33 (*Hordeum vulgare*)
	LOC8068183	-0.63	-0.41	-1.17	Transcription factor MYB108 (*Arabidopsis thaliana*)
	LOC8069712	-0.55	-0.18	-1.11	Transcription factor PCL1 (*Oryza sativa* subsp. *japonica*)
bZIP	LOC110433340	1.15	-0.28	3.68	G-box-binding factor 3 (*Arabidopsis thaliana*)
	LOC8059898	0.60	0.02	3.03	bZIP transcription factor
	LOC8075201	0.90	-0.35	3.01	Abscisic acid-insensitive-5-like protein 6 (*Arabidopsis thaliana*)
	LOC8058560	0.08	-0.02	2.95	G-box-binding factor 3 (*Arabidopsis thaliana*)
	LOC8077703	0.80	0.17	2.47	Basic leucine zipper 43 (*Arabidopsis thaliana*)
	LOC8077354	0.09	0.28	1.83	bZIP transcription factor TRAB1 (*Oryza sativa* subsp. *japonica*)
	LOC8082252	-0.07	0.31	1.77	Transcription factor HY5 (*Arabidopsis thaliana*)
	LOC8084187	0.24	-0.13	1.36	bZIP transcription factor
	LOC8062618	-0.20	0.16	1.19	Transcription factor RF2b (*Oryza sativa* subsp. *japonica*)
	LOC110437038	0.18	-0.25	1.08	bZIP transcription factor TRAB1 (*Oryza sativa* subsp. *japonica*)
bHLH	LOC8054916	0.77	0.72	1.52	Transcription factor bHLH144 (*Arabidopsis thaliana*)
	LOC8081697	0.28	-0.10	1.10	Transcription factor bHLH35 (*Arabidopsis thaliana*)
	LOC8080455	0.64	0.41	1.01	Transcription factor bHLH54 (*Arabidopsis thaliana*)
	LOC8055095	-0.50	-0.44	-1.82	Transcription factor bHLH19 (*Arabidopsis thaliana*)
	LOC8054487	-0.39	-0.55	-1.44	Transcription factor LHW (*Arabidopsis thaliana*)
	LOC8068593	-0.78	-0.81	-1.40	Transcription factor bHLH155 (*Arabidopsis thaliana*)
	LOC8073915	0.28	1.04	0.05	Transcription factor bHLH111 (*Arabidopsis thaliana*)
	LOC8055721	1.20	0.78	0.87	Putative transcription factor bHLH041 (*Arabidopsis thaliana*)

a, represents fold changes calculated from log_2_ of RPKM values of experimental samples (100/100, uniform salinity; 0-0/200 and 200-0/200, non-saline and high saline root sides of non-uniform salinity treatments, respectively) vs those of CK.

### Validation of DEGs via q-PCR

**Fig 5:**
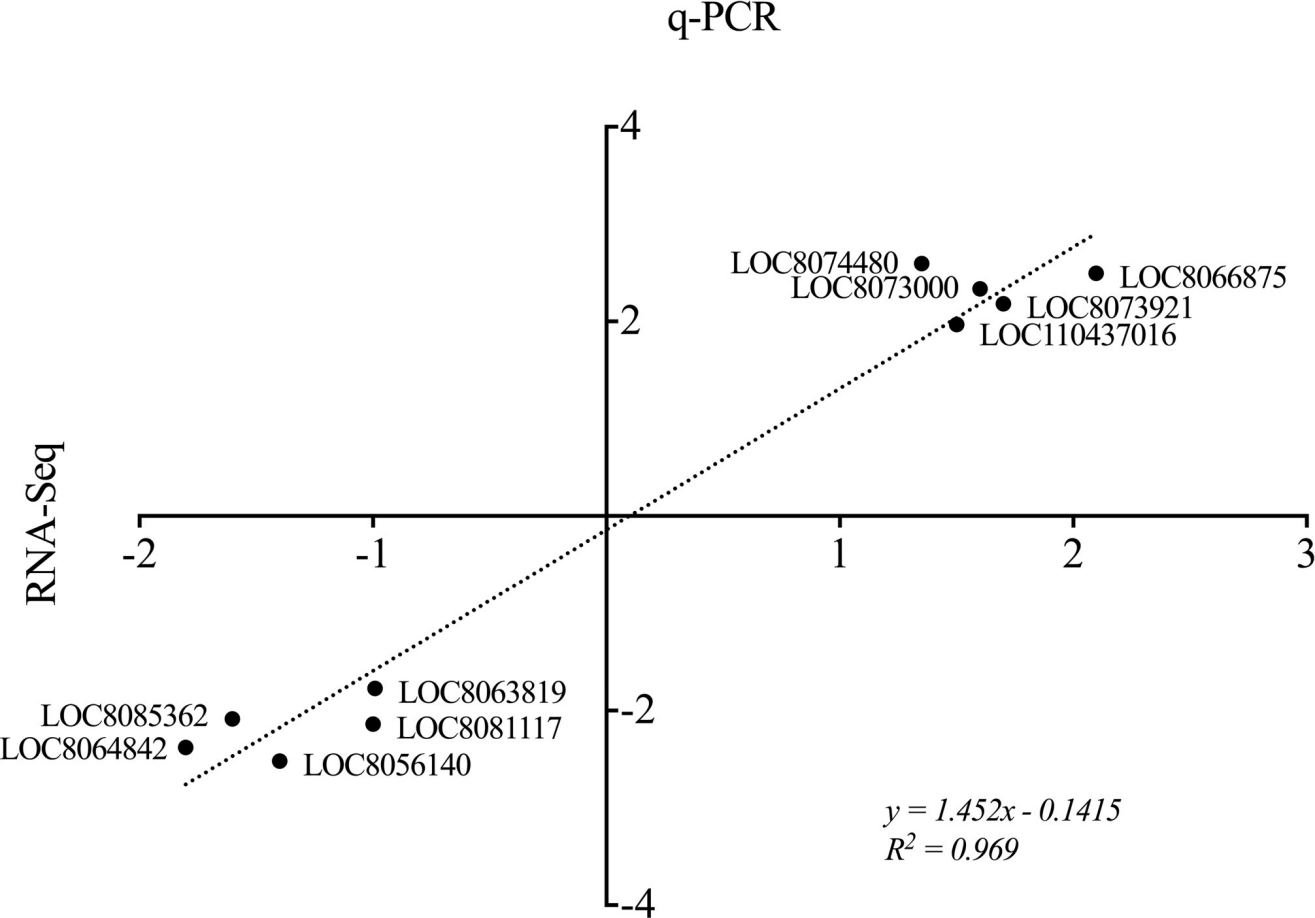
Validation of the expression profiles of 20 randomly chosen DEGs obtained from RNA-seq via quantitative PCR (q-PCR). Shown are the fold changes of the log_2_ values resulting from RNA-seq, and the q-PCR results fit a linear regression (*y = 1*.*425x-0*.*1415*, *R*^*2*^ *= 0*.*969*).

The expression patterns of DEGs identified by RNA-seq were validated for 20 randomly chosen DEGs that were expressed in the leaves or roots and that exhibited different expression profiles in the different stress treatments. The qRT-PCR results showed that the up- or downregulated expression patterns of the 20 genes were similar to the results obtained by RNA-seq. The results of qRT-PCR were highly consistent with those of RNA-seq and fit a linear correlation, with a relative *R*^*2*^ value of 0.969 (**Fig 5**).

## Discussion

The spatial distribution of salinity is rarely uniform in soils [36]. Compared with those focusing on uniform salinity conditions, studies related to nonuniform salinity conditions are lacking. Therefore, determining how plants, especially crop plants, react in nonuniform salinity situations to avoid salt damage will complete our lack of global knowledge of salt tolerance mechanisms [37]. The present study attempts to reveal the underlying DEGs that confer adaptability to rhizospheric nonuniform salinity distributions to sorghum seedlings.

### Phenotypic adaptations revealed by physiological measurements

In the present study, compared with that under uniformly distributed root zone salinity, the physiological status of sorghum seedlings under nonuniformly distributed root zone salinity was largely improved (**Fig 1**). Under uniform salt stress conditions, the seedlings had greater Na^+^ contents in both the leaves and roots than did their counterparts under nonuniform salt stress conditions. However, most Na^+^ was present in the high-saline root side in the nonuniform salinity treatment. This demonstrated a low degree of salt stress imposed on the seedlings under the nonuniform salt stress conditions. Our finding was supported by Kong et al.’s report on cotton in a similar split-root system [38]. Moreover, the sorghum seedlings were subjected to a low level of excess salt that caused oxidative stress and osmotic stress under nonuniform salt stress conditions, as SOD, POD, and CAT activities as well as Pro and GSH contents increased under such conditions. These observations were confirmed in alfalfa by Xiong et al. [39]. With respect to photosynthesis, we noted that, compared with those in the uniform saline group, the net photosynthesis, chlorophyll content, and stomatal conductance in the nonuniform saline group increased, which was in line with the results of Chen et al.’s study [36]. Furthermore, the transpiration rate was reduced as a result of increased levels of salt stress [40, 41]. The present study showed that sorghum seedlings treated with uniform salt stress suffered from more severe salt injury than did those treated with nonuniform salt stress because their transpiration rate was not diminished as much as that of the uniform salt stress-treated seedlings was. These physiological adaptations could explain the increased biomass of the sorghum seedlings 2 weeks after they were subjected to nonuniform salinity stress. These results were consistent with those of previous studies, suggesting that an adaptation of genic regulation at the transcriptional level underlies the phenotypic adaptation to uneven salinity dispersal around roots, which accounts for the growth improvement under nonuniform salt stress conditions. Using the RNA-seq technique, we profiled the gene expression patterns of sorghum seedlings under both uniform and nonuniform salt stress conditions and detected DEGs related to excess Na^+^/ROS detoxication, salt stress perception/response, photosynthesis, and nutrient/water uptake.

### DEGs associated with Na^+^ transport under uniform and nonuniform salt stress conditions

To combat salt injury caused by unnecessary Na^+^, plant cells have developed physiological approaches to equilibrate ion homeostasis. For example, the SOS1, SOS2, and SOS3 pathways translocate excess Na^+^ from the cytosol to the intercellular space [42]. NHX genes encode proteins that sequester extra cytosolic Na^+^ by moving it to vacuoles to evade ionic stress triggered by salinity [43]. Rebalancing the Na^+^/K^+^ ratio to a low value under salt stress is crucial for plants to survive high salinity [44]. HKT1 along with a group of K^+^ transporter genes predominates over the maintenance of such a minimal Na^+^/K^+^ ratio [44]. In addition, ABC transporters modulate ion transmembrane relocation as well as ABA under salt stress and thus increase salt resistance [45]. The expression levels of SOS 2 and ABC transporter genes as well as a number of genes encoding Na^+^ and K^+^ transporters were upregulated in the leaves of plants under nonuniform salinity conditions (**Table 1**). However, the expression levels of these identified DEGs were nearly unchanged under uniform salinity conditions. The moderate Na^+^ concentration in the leaves and improved leaf growth under nonuniform salt stress could be attributed to the aforementioned findings of the present study. Additionally, the increased expression levels of the genes identified in our transcriptomic data demonstrated that the high salinity-stressed root side of the nonuniformly distributed-Na^+^-treated seedlings could stimulate the expression of vital genes participating in salt stress defensive pathways and accordingly boost salt tolerance [6].

### DEGs associated with photosynthesis under uniform and nonuniform salinity stress

The expression of genes associated with photosynthesis-related pathways are suppressed under salt stress [6, 46]. Our results showed that the expression of genes involved in photosynthesis was inhibited under uniform salinity stress compared with nonuniform salinity stress (**Table 1**). For instance, the expression levels of the gene coding for the electron donor in the photosynthetic chain, ferredoxin, and the gene coding for chlorophyll a/b binding protein 3 of light-harvesting complex II were downregulated under uniform Na^+^ stress. Additionally, the expression of the gene coding for mitogen-activated protein kinase 5 (MAPK5), which is involved in the stress signaling pathway as well as stress adaptations involving photosynthesis, was downregulated [42, 47]. In contrast, the expression of these genes increased in the nonuniform salinity (0 mmol·L^-1^ and 200 mmol·L^-1^ NaCl)-treated sorghum seedlings. This might explain the improved photosynthesis performance under the nonuniform salt stress conditions.

### DEGs associated with osmotic and oxidative stress under uniform and nonuniform salinity stress conditions

Salt stress causes cellular water loss and produces ROS, which disrupt biochemical reactions [48, 49]. Chitinase is generally a useful target related to plant reactions against salt stress, and its increased expression level confers a higher salt resistance to plants [50, 51]. Similarly, increased expression of phospholipase D1 decreases osmotic stress [52]. Phospholipase D1 can alleviate osmotic stress by mediating phosphatidic acid (PA) synthesis [53]. Our results showed that the expression levels of chitinase and phospholipase D1 increased by 3.05- and 1.50-fold, respectively, under nonuniform salinity stress, whereas the expression levels of both genes were downregulated in the seedlings under uniform salinity stress (**Table 1**). Our results showed that the expression levels of the genes coding for POD, CAT, and SOD also increased by up to 7.39-fold, 2.66-fold, and 1.58-fold, respectively, under nonuniform salt stress conditions compared with uniform salt stress conditions, the latter under which the POD, CAT, and SOD expression levels largely decreased (**Table 1**). Together, these phenomena might contribute to the relieved salt stress and to the improved growth, which was reflected by the increase in biomass of the sorghum seedlings at 2 weeks after the stress was imposed under nonuniform salinity conditions.

### DEGs associated with phytohormones under uni- and nonuniform salinity stress

Among the plant hormones, ABA is a pivotal participant that mediates several responses to water deficit induced by salt and drought stress [54]. ABA synthesis depends on the rate-limiting oxidization of 9’-cis-neoxanthin to the intermediate precursor xanthoxin by 9’-cis-epoxycarotenoid dioxygenase (NCED) [55]. In the present study, the expression level of the gene coding for NCED increased by 3.62-fold in the leaves under nonuniform salt stress conditions (**Table 1 and Table 2**). However, the expression increased under uniform salt stress conditions, although its expression fold change was less than that under nonuniform salt stress conditions. In the roots, the expression of the NCED gene increased by 3.99-fold in the high-Na^+^ root side under nonuniform saline conditions. Additionally, our results also revealed the activation of a number of genes involved in ABA-mediated signaling pathways in the high-saline root side of seedlings exposed to nonuniform salinity conditions, whereas their expression levels in the roots under uniform salinity conditions were downregulated, which differed from the findings in Kong et al.’s [6] report on signaling pathways activated within the nonsaline root side. Moreover, we detected several gene transcripts for additional phytohormones associated with high salinity-induced stress (**Table 1**). For example, the transcript levels of indole-3-acetaldehyde oxidase (IADO) for indole-3-acetic acid (IAA) synthesis were upregulated by 1.57-fold under nonuniform salt stress conditions. It has been reported that the biosynthesis of IAA can be directed through the redundant indole-3-acetaldehyde (IAD) pathway, which depends on IADO [56, 57]. The activity of IADO increased by approximately 5-fold in an IAA overexpression Arabidopsis mutant [58]. Furthermore, we detected an increase in gene transcripts for anthranilate synthase component II; these transcripts could interact with the BEM46 protein, which may be associated with auxin synthesis [59]. Additionally, IAA is coupled with the salt stress defense response, although the basic mechanism remains unknown [13]. Newly reported transcriptomic research has shown that transcripts involved in the ABA and auxin signaling pathways were upregulated as a result of osmotic and salt stress [60], and the exogenous application of auxin-like chemicals could ease injuries caused by water deficit [61]. Along with the increase in the abundance of transcripts related to auxin biosynthesis, an unknown transcript related to ethylene synthesis was detected in our transcriptomic data (**Table 1, Table 2**). Both auxin and ethylene respond to salt stress and promote plant growth [62]. This might contribute to the improved growth of sorghum seedlings under nonuniform Na^+^ stress conditions. Interestingly, our transcriptomic data revealed gene transcripts that are related to SA and JA biosynthesis and that could act in response to salt stress [63, 64]. Foliar sprays of these two compounds can increase tolerance to abiotic stress, such as salt stress [65]. In the present study, the gene transcript for 12-oxo-phytodienoic acid reductase was upregulated under nonuniform salt stress conditions compared with uniform salt stress conditions (**Table 1**); this gene responds to JA [66]. In our study, compared with that under uniform salt stress, the expression of a notable gene transcript encoding MAPK5 under nonuniform salt stress was upregulated by approximately 9.5-fold (**Table 1**). The expression of this gene was triggered in response to salt stress is was associated with the biosynthesis of SA [67, 68]. The expression of chitin elicitor receptor kinase 1 (CERK1), which is associated with SA [69], was upregulated under nonuniform salinity stress but remained almost unchanged under uniform salinity stress in the present study. These three genes could be considered targets for investigating salt tolerance mechanisms.

### DEGs associated with water and nutrient transport under uniform and nonuniform salinity stress conditions

Roots are an important plant organs that nourish entire plants by taking up water and essential minerals. In addition, the roots are the very first location at which plants encounter osmotic stress caused by salt or drought. This produces changes in the expression levels and abundance of root-specific aquaporin genes and proteins and mineral transporter genes and proteins, respectively [19]. The present study resulted in the identification of 22 PIP and TIP transcripts differentially expressed between the nonuniform and uniform salinity treatments (**Table 3**). The downregulation of PIPs is highly correlated with strong adaptive responses to water deficit conditions in roots, though contrasting observations have been reported in different plant species [70, 71]. The role of TIPs in roots was highlighted in the study of chickpea under water deficit conditions, in which the expression of most of the TIPs was downregulated, with a few exceptions [72]. Generally, the expression of regulatory aquaporins in response to stress is not universal among different aquaporins [73]. However, through the complex regulatory network of aquaporin expression, plants can become tolerant to water stress caused by high salt and drought [12]. Our results showed that most PIP and TIP transcription was inhibited under both salinity conditions but remained almost unchanged under nonsalinity conditions, suggesting a probable transcriptomic tuning of aquaporins to maintain water uptake through the nonsaline rhizosphere of seedlings in nonuniform saline environments. Furthermore, we detected 12, 19, and 10 mineral transporter genes whose expression was up- or downregulated in response to nitrate, K^+^, and phosphate (**Table 4**). Interestingly, the mineral transporter genes whose expression was upregulated in the nonsaline root side under nonuniform salinity conditions outnumbered those in the high-saline root side under nonuniform salinity conditions as well as the mineral transporter genes under uniform salinity conditions, which was in agreement with the results of Kong et al.’s [6] report. The relieved water and mineral uptake could thus contribute to the better performance of the seedlings under the nonuniform saline environment.

### DEGs associated with salt stress-responsive TFs under uniform and nonuniform salinity stress conditions

TFs serve as molecular toggles to trigger stress-induced gene expression and thus play a substantial role in modulating the adaptative pathways of plants against salt stress [74, 75]. A variety of TFs have been described in terms of their roles in the salt stress response. TFs that likely mediate salt stress signal transduction and downstream gene expression include MYB/MYCs, ERFs, bHLHs, bZIPs, NACs, WRKYs, and so forth [76, 77]. In this study, our transcriptomic data revealed a number of differentially expressed TFs in both the leaves and the roots (**Table 5**). For example, our results showed that NACs, WRKYs, ERFs, zinc finger proteins, basic region leucine zippers, and PLATZs were highly induced in the leaves of plants subjected to nonuniformly distributed salinity in their rhizosphere. Moreover, the expression levels of TFs were upregulated by up to 6.24-fold under nonuniform salinity conditions, whereas those of the TFs were downregulated or remained unaffected under uniform salinity conditions. In the roots, we detected 61 and 20 TFs whose expression was upregulated and downregulated, respectively, in the high-Na^+^ root side, while four and one upregulated and downregulated TFs, respectively, were detected in the salt-free root side. These findings were consistent with those of a study by Kong et al. on cotton under nonuniformly distributed salinity conditions [6] and suggested roles of the TFs in stress-induced adaptation pathways as discussed previously [75]; moreover, these phenomena could explain the relief of salt stress by nonuniform salinity conditions to a lower level than that afforded by uniform salinity conditions.

## Conclusions

Soil salinity is an important issue that affects yields. Salinity is not evenly distributed throughout soils. However, the nonuniform distribution of soil salinity can, to a certain extent, alleviate salt damage and thus relieve the inhibition of plant growth. Underlying the apparent improvement of physiological performance under nonuniformly distributed salinity conditions is the molecular adaptation of plants fueled by the differential expression of stress-responsive gene transcripts under such stress conditions. In the present research, a moderately salt-tolerant crop species (sorghum) in a split-root system was used to elucidate this molecular mechanism at the transcriptomic level. Our transcriptomic analysis revealed that, under such stress conditions, sorghum maintained an improved growth pattern via a series of DEGs that play essential roles in the pathways of photosynthesis, the antioxidative defense system, ionic homeostasis regulation, phytohormone signal transduction, and TF networks. These pathways were initiated by high-saline regions of the rhizosphere subjected to nonuniform soil salinity conditions. Additional studies are needed to fully decipher the mechanism to refresh our understanding of how sorghum copes with nonuniform salinity stress, which would be helpful for the development of new farming strategies to alleviate salt stress by manually establishing such an environment with spatially inconsistent salt distribution in the soil.

## Supporting information

S1 Table Primer sequences of differentially expressed genes for q-PCR validation. (DOCX)

S2 Table Statistics of RNA-Seq data. (DOCX)
