# Supplementary material for: Heterogeneous root zone salinity mitigates salt injury to Sorghum bicolor (L.) Moench in a split-root system
Source: PLoS One. 2019 Dec 30;14(12):e0227020. doi: 10.1371/journal.pone.0227020 (PMC6936808; doi:10.1371/journal.pone.0227020)
Supplement: S1 Table — (DOCX) [file pone.0227020.s001.docx]

**S1 Table** Primer sequences of differentially expressed genes for q-PCR validation

| Locus name | Forward/reverse | Primer sequence |
| --- | --- | --- |
| LOC110436378 (Actin 1) | F | CATCCCTCAGCACCTTCCAG |
|  | R | TAATGGCTCCTCTCGGCTTG |
| LOC8074727 | F | TCGGATGATTTGCGGCACTA |
|  | R | AGATTCCGTGTCGGTTTCCC |
| LOC8069827 | F | AGGTCTCAAGTCTCAACTCAACA |
|  | R | CCACAAGGAGGCAGGCTAT |
| LOC8062360 | F | GGATGGAGGAGTAGCCGAATA |
|  | R | CCGATACAGAGATGGTGGTGG |
| LOC8056001 | F | TACGCCTGATCCAATCCACC |
|  | R | AGTGACCACGACGAGACAGG |
| LOC8056861 | F | AAGCATCCAACAACAAGGCTC |
|  | R | AAGTAGTAGAAGTCGAGGCGG |
| LOC8064911 | F | ACAGGGCTCTCTTGCCATTT |
|  | R | CTTCATTTCGCGCTGCACTA |
| LOC8063814 | F | TTGTTGATCGAGCGAGCTGT |
|  | R | ACGTCCTGGTAGAAGTTGCC |
| LOC8080433 | F | GGAAGGACATCTTCATGTACCGC |
|  | R | TGGAACTCGTCGCTCACGAT |
| LOC8075100 | F | CAAAGATGCCCGATTGCGTA |
|  | R | ACCGCAGCTAACATTTGGGA |
| LOC8082122 | F | CGGTCGATCCATCCATCCTT |
|  | R | TCTTCGAGCAGCAGTCTTGT |
| LOC8074480 | F | TCAACAGGTTCTCCTCCGTC |
|  | R | TGATCGAGATGCTAACAAGAGGT |
| LOC8066875 | F | GTGCAAGGCATCACCATCCT |
|  | R | CGTCGCCAGACACGATGTA |
| LOC8073000 | F | CACCAGTGCATGATCCGT |
|  | R | CTCTGACTACAAGAGAGTACAAGT |
| LOC8073921 | F | GACTCAAGAGCGAGTTCCAGT |
|  | R | CTCTCATCCTCCATGATCGCC |
| LOC110437016 | F | TCGCTCTCAAGCTTCTCCATC |
|  | R | ACGCTAACCACCATCGTTTTC |
| LOC8063819 | F | TGACAAGTCTCTTCTGGATGACG |
|  | R | ATGAGGCAGTGGAAGTGGTG |
| LOC8085362 | F | AGTGTGCGCTTGGATGATTG |
|  | R | CAGACCGCAGAAGGAACAAG |
| LOC8081117 | F | GTTGTTGCGAGAGACACACA |
|  | R | ATATACAGAGGATTCGAGCGTG |
| LOC8064842 | F | GGACATGGTGGTCACTGGAA |
|  | R | GGCCGTCCAAGTTCCTACATT |
| LOC8056140 | F | TAGCCAACAGGAAGCAGACG |
|  | R | TGCTCACTCCAACCTCTCAC |
