# Supplementary material for: Heterogeneous root zone salinity mitigates salt injury to Sorghum bicolor (L.) Moench in a split-root system
Source: PLoS One. 2019 Dec 30;14(12):e0227020. doi: 10.1371/journal.pone.0227020 (PMC6936808; doi:10.1371/journal.pone.0227020)
Supplement: S2 Table — (DOCX) [file pone.0227020.s002.docx]

| Sample | Treatment | Replication | Total clean reads | Mapped reads (%) | Uniq mapped reads (%) | GC content (%) | ≥ Q30 (%) |
| --- | --- | --- | --- | --- | --- | --- | --- |
| Leaf | CK | 1 | 56,817,084 | 47,213,709 (83.10%) | 46,161,457 (81.25%) | 53.99% | 91.40% |
|  |  | 2 | 57,714,392 | 47,704,251 (82.66%) | 46,649,082 (80.83%) | 54.16% | 90.85% |
|  |  | 3 | 53,365,218 | 43,946,367 (82.35%) | 43,051,477 (80.67%) | 54.35% | 90.93% |
|  | 100/100 | 1 | 48,871,428 | 40,460,948 (82.79%) | 39,705,161 (81.24%) | 53.95% | 90.86% |
|  |  | 2 | 53,093,494 | 43,840,128 (82.57%) | 43,078,095 (81.14%) | 54.28% | 90.99% |
|  |  | 3 | 58,751,478 | 48,464,135 (82.49%) | 47,595,324 (81.01%) | 54.77% | 90.88% |
|  | 0/200 | 1 | 59,462,832 | 48,999,307 (82.40%) | 48,088,399 (80.87%) | 54.31% | 90.59% |
|  |  | 2 | 56,492,692 | 46,681,527 (82.63%) | 45,937,804 (81.32%) | 55.10% | 91.05% |
|  |  | 3 | 62,572,146 | 51,608,050 (82.48%) | 50,715,001 (81.05%) | 54.72% | 90.75% |
| Root | CK | 1 | 47,807,210 | 38,153,034 (79.81%) | 37,473,788 (78.39%) | 55.37% | 90.29% |
|  |  | 2 | 52,443,966 | 41,653,912 (79.43%) | 40,836,738 (77.87%) | 55.24% | 90.59% |
|  |  | 3 | 48,692,952 | 38,579,000 (79.23%) | 37,838,659 (77.71%) | 55.11% | 90.25% |
|  | 100-100/100 | 1 | 49,951,256 | 39,651,878 (79.38%) | 38,992,695 (78.06%) | 54.21% | 90.60% |
|  |  | 2 | 45,634,682 | 35,809,387 (78.47%) | 35,103,515 (76.92%) | 55.58% | 90.21% |
|  |  | 3 | 48,116,340 | 38,264,250 (79.52%) | 37,557,500 (78.06%) | 54.84% | 90.17% |
|  | 0-0/200 | 1 | 46,585,614 | 36,660,747 (78.70%) | 36,004,592 (77.29%) | 54.95% | 90.37% |
|  |  | 2 | 52,544,478 | 41,533,458 (79.04%) | 40,572,652 (77.22%) | 55.01% | 90.46% |
|  |  | 3 | 47,186,752 | 37,288,866 (79.02%) | 36,571,686 (77.50%) | 55.65% | 90.54% |
|  | 200-0/200 | 1 | 48,176,454 | 38,691,932 (80.31%) | 37,902,405 (78.67%) | 54.63% | 90.61% |
|  |  | 2 | 48,006,478 | 37,950,294 (79.05%) | 37,379,006 (77.86%) | 55.32% | 90.35% |
|  |  | 3 | 52,442,386 | 40,309,284 (76.86%) | 39,246,185 (74.84%) | 55.50% | 90.55% |
|  | | | | | | | |

**S2** **Table** Statistics of RNA-Seq data
